# Brain Vascular Microenvironments in Cancer Metastasis

**DOI:** 10.3390/biom12030401

**Published:** 2022-03-04

**Authors:** Lucas E. Tobar, Rae H. Farnsworth, Steven A. Stacker

**Affiliations:** 1 Peter MacCallum Cancer Centre, Melbourne, VIC 3000, Australia; lucas.tobar@petermac.org (L.E.T.); rae.farnsworth@petermac.org (R.H.F.); 2 Sir Peter MacCallum Department of Oncology, University of Melbourne, Parkville, VIC 3010, Australia; 3 Department of Surgery, Royal Melbourne Hospital, University of Melbourne, Parkville, VIC 3050, Australia

**Keywords:** metastasis, brain, leptomeninges, blood vessels, lymphatic vessels, stroma, microenvironment

## Abstract

Primary tumours, particularly from major solid organs, are able to disseminate into the blood and lymphatic system and spread to distant sites. These secondary metastases to other major organs are the most lethal aspect of cancer, accounting for the majority of cancer deaths. The brain is a frequent site of metastasis, and brain metastases are often fatal due to the critical role of the nervous system and the limited options for treatment, including surgery. This creates a need to further understand the complex cell and molecular biology associated with the establishment of brain metastasis, including the changes to the environment of the brain to enable the arrival and growth of tumour cells. Local changes in the vascular network, immune system and stromal components all have the potential to recruit and foster metastatic tumour cells. This review summarises our current understanding of brain vascular microenvironments, fluid circulation and drainage in the context of brain metastases, as well as commenting on current cutting-edge experimental approaches used to investigate changes in vascular environments and alterations in specialised subsets of blood and lymphatic vessel cells during cancer spread to the brain.

## 1. Introduction

Cancer arises when acquired or inherited mutations in the cells of the body result in uncontrolled cellular proliferation. In the case of solid tumours, the most lethal attribute of cancer is the ability of tumour cells to escape the primary tumour and metastasise via blood and lymphatic vessels to distant organs. Assessment of the number and location of metastatic lesions in a patient forms a critical part of disease staging and dictates both prognosis and treatment options [1]. Metastatic lesions in the brain that arise from distant primary solid tumours, such as lung, breast and melanoma, result in a particularly poor prognosis [2]. It has been speculated that the frequency of brain metastasis diagnoses has steadily risen over time due both to ongoing improvements in imaging and detection technologies that allow for greater detection rates of brain metastases in asymptomatic patients, and improvements in systematic disease treatments that prolong survival and allow more time for brain lesions to develop [3,4]. A population-based study has shown that, cumulatively, 12% of patients with metastatic disease originating from over 27 different primary cancer types were found to have brain metastases upon primary cancer diagnosis, with a median survival of approximately five months [5]. The study also showed elevated incidence of brain metastases in patients with melanoma (28%) and lung cancers (16–26%) relative to the other tumour types examined. The increasing incidence of brain metastases and the associated poor prognosis thus illustrates the importance of developing a more detailed understanding of their aetiology and biology that may elucidate novel targets for therapy.

The brain is a vital, complex, and intricately compartmentalised organ, containing equally complex and specialised vascular microenvironments in each of its distinct anatomical regions. Perhaps the most specialised and fundamental component of the brain vasculature is the blood–brain barrier (BBB). The BBB, a key feature of the vessels penetrating into the parenchymal tissue, refers to the tightly regulated assembly of cells including the endothelial cells, pericytes, vascular smooth muscles cells, and astrocytes that are collectively known as the neurovascular unit (NVU) [6]. Endothelial cells are adhered to each other via tight junctions and are surrounded by the endothelial basement membrane (BM). Perivascular supporting cells such as pericytes and vascular smooth muscle cells, collectively referred to as mural cells, together with astrocytic foot-processes further encapsulate the endothelial cells, anchored through an astrocytic basement membrane. This multi-layered barrier of cells and basement membrane only allows molecules of molecular weight less than 400–500 Da to passively diffuse into the parenchyma, working in synergy to regulate molecular and cellular trafficking across the brain vasculature, and thus maintaining homeostasis of the central nervous system (CNS) [7].

Early tumour cell seeding as well as tumour-derived extracellular vesicles or circulating factors from distant primary tumours will often shape the microenvironment in secondary sites such as the brain before tumour cell colonisation, creating a favourable environment for secondary tumour establishment termed the pre-metastatic niche [8,9,10,11]. Cells of the NVU, together with immune cells of both myeloid and lymphoid lineages, can thus be hijacked by infiltrating tumour cells, leading to BBB breakdown and creating a metastatic niche that supports the tumour’s initial establishment and survival as well as its ongoing growth and proliferation [12,13]. Notably, the unique vascular microarchitecture of the brain influences mechanisms and patterns of metastatic colonisation. These processes play a key role in the metastatic cascade from a primary to an established secondary tumour yet remain poorly characterised.

In this review, we discuss the current understanding of brain vascular microenvironments, fluid circulation and drainage in the context of brain metastases. We describe the current understanding of the cellular components of the metastatic tumour microenvironment in the brain, with a specific emphasis on tumour–vasculature interactions during the early events of tumour cell seeding in the brain and the formation of established metastatic lesions. In addition, we comment on current cutting-edge experimental approaches used to support our understanding of the tumour–vascular interactions in brain metastasis.

## 2. Central Nervous System Vasculature and Anatomy

### 2.1. CNS Anatomy and Blood Supply

**Figure 1:**
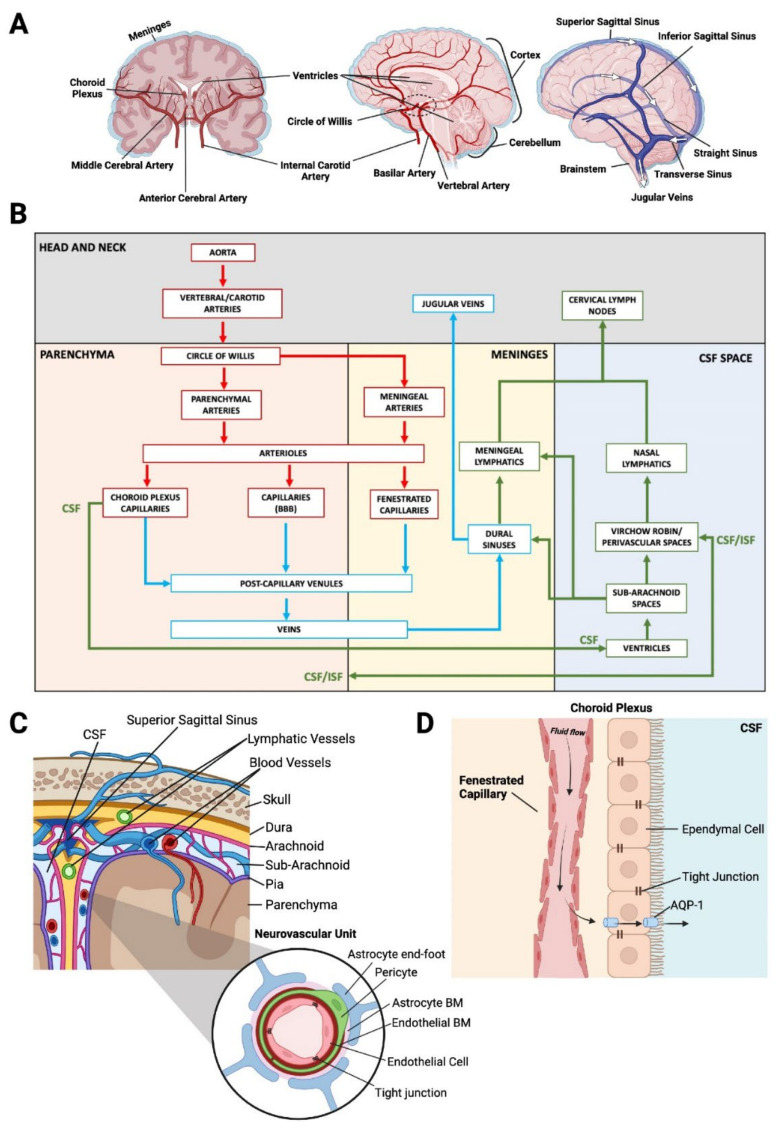
(**A**)—Coronal cross-section of brain arterial supply (left), sagittal cross-section of brain arterial supply (middle), sagittal view of brain venous system (right). (**B**)—2D roadmap of blood and fluid circulation within the brain. The Aorta gives rise to the vertebral and carotid arteries, where at the circle of Willis, smaller parenchymal (anterior, middle and posterior cerebral arteries) and meningeal arteries branch off to supply different regions of the brain. Parenchymal arteries branch into arterioles and further into capillaries, all of which display blood–brain barrier (BBB) phenotypes. Capillaries coalesce into post capillary venules, which go on to form larger veins. Veins in the brain drain into the dural sinuses situated in the meninges, where molecules are sampled by the meningeal lymphatics before draining into the jugular veins. Importantly, blood vessels within the Dura are fenestrated, and thus lack the abundance of tight junctions typical of endothelial cells of the BBB. The cerebrospinal fluid (CSF) fluid flow, also referred to as the glymphatic system, begins with the production of CSF from fenestrated capillaries (no BBB) of the choroid plexus into the ventricles. CSF flows from the ventricles to the sub-arachnoid spaces, and further into Virchow–Robin and perivascular spaces which form between the basement membrane layers of endothelium and surrounding astrocytes. Virchow–Robin spaces are sites of fluid exchange between the interstitial fluid (ISF) of the parenchyma and the CSF space. The nasal lymphatic system receives drainage from the CSF space, and together with the meningeal lymphatics, drains into the cervical lymph nodes. Red arrows indicate arterial blood flow, blue indicates venous blood flow, green arrows indicate “glymphatic flow” which includes lymphatic fluid, CSF, and ISF. (**C**)—Coronal view of the surface of the cortex. The three meningeal layers are located between the parenchyma and skull, with the most superficial layer being the dura mater, followed by the arachnoid mater, sub-arachnoid space and finally, the pia mater. The dura mater contains a recently discovered network of lymphatic vessels and the sub-arachnoid space is host to a complex network of veins and arteries at the cortical–meningeal surface. The Neurovascular unit is comprised of select cells creating a tightly regulated system of molecular transport between the blood and the parenchyma (BBB). Endothelial cells are adhered to each other via tight junctions and are surrounded by endothelial basement membrane (BM). Pericytes and astrocytic foot processes further encapsulate the endothelial cells, anchored through an astrocytic basement membrane. (**D**)—Fluid flow within the choroid plexus. Ependymal cells of the choroid plexus create a blood–CSF barrier due to the presence of tight junctions. Passage of fluid extravasated from the fenestrated capillaries within the choroid plexus into the CSF space is regulated via transport through aquaporins such as AQP1.

The brain is anatomically complex, containing several distinct compartments including the parenchyma, ventricles, brainstem, and meningeal layers at the cortical surface. Each of these compartments houses phenotypically unique vascular environments, and all depend on a continuous supply of blood from a highly integrated network of penetrating blood vessels arising from the neck region (Figure 1A,B). The anterior circulation of the brain is provided by the internal carotid arteries, whereas the posterior circulation is supplied by the vertebral arteries which join to form the basilar artery that also supplies the brainstem and cerebellum (vertebral–basilar circulation) [14]. The posterior and anterior circulation systems are connected via communicating arteries that form the circle of Willis, which exists as a compensatory mechanism in case blood flow is decreased in a particular system [15]. The anterior and middle cerebral arteries which branch from the internal carotid arteries, together with the posterior cerebral artery (supplied by the posterior circulation) supply the deep regions of the brain through penetrating branches into the cortex.

The major arteries and penetrating blood vessels of the brain parenchyma contain specialized BBB endothelial cells that create a highly selective semipermeable border due to the abundance of tight junctions that adhere neighbouring cells to each other, forming a tight barrier [6] (Figure 1C). This in turn prevents most molecules in the circulation from non-selectively traversing the endothelial cell layers into the interstitial fluid of the brain that surrounds the parenchymal cells. Typically, only molecules of molecular weight less than 400–500 Da are able to passively diffuse across the BBB [7]. Admission of any other molecules is selectively enabled by substrate-specific molecular transporters and channels. Even essential nutrients such as glucose and amino acids are often too large to be transported freely across the BBB. Transporters such as glucose transporter 1 (GLUT1), and large neutral amino acid transporter 1 (LAT1) are therefore required to transport key nutrients such as glucose and amino acids across the BBB and into the neuronal tissue to meet the metabolic needs of the parenchymal cells [16]. Other supporting cells, such as astrocytes and pericytes, wrap around the blood vessels within the NVU and throughout the brain, further regulating the transport of select molecules across the BBB and maintaining the integrity of the NVU [16].

The meninges, which encapsulate the brain, are composed of three membranous layers known as the dura, arachnoid and pia mater, each unique in its contributions to the vasculature present at the cortical surface (Figure 1C) [17]. The dura is the outermost layer and contains fenestrated blood vessels that lack the typical tight junctions present in the BBB [18]. The external carotid artery is an additional artery of significance for the brain; specifically, one of its largest branches, the middle meningeal artery, supplies more than two thirds of the cranial dura [19]. The dura is also supplied anteriorly by branches of the ophthalmic artery that originate from the ICA, as well as posteriorly from vertebral artery branches, further connecting the vascular network around the cranium [20]. The arachnoid and pia mater are together referred to as the leptomeninges. Importantly, the arachnoid layer of cells contains tight junctions and efflux pumps, acting as a barrier between the dura and the cerebrospinal fluid (CSF)-filled sub-arachnoid space sitting between the arachnoid and pia mater [21]. This area has a primary function of cushioning the underlying brain parenchyma and is also host to the large arteries and veins that carry blood into and out of the brain [22].

### 2.2. Arteriovenous Zonation

The vasculature of the brain, as with other organs, branches hierarchically from larger vessels down to the smaller capillaries where most of the molecular and cellular exchange occurs between circulation and the brain parenchyma. Anatomically, larger arteries at the cortical–meningeal surface will branch into arterioles that penetrate into the brain parenchyma, maintaining the characteristic phenotypical features of the blood–brain barrier (Figure 1B). Arterioles then branch into capillaries, which in turn drain into post-capillary venules [23,24]. Venules then carry deoxygenated blood out of the brain parenchyma into large venous sinuses located in the dura (see Section 2.3).

Cellular zonation refers to the progressive phenotypical differences and changes observed in classes of cells along an anatomical axis [25]. In endothelial cells, cellular zonation occurs across an arteriovenous axis. While a handful of regulators of arterial and venous specification and function have been described [26,27], more detailed characterisation of vascular heterogeneity at the transcriptomic level is only just beginning. In the brain vasculature, arteriovenous zonation has been illustrated with particular clarity in a landmark single-cell RNA sequencing (scRNA-seq) study of endothelial and mural cells of the mouse brain [28]. Gradually changing gene expression was evident when the cells were grouped into clusters based on transcriptional similarity, with transcriptomic signatures indicative of arterial, capillary and venous identity suggested to be in a seamless continuum of transcriptional states from arterial to venous. Known arterial markers used to identify arterial endothelial cells included *Bmx*, *Efnb2*, *Vegfc* and *Sema3g*, and venous markers included *Nrf2*. Gradual changes in expression of genes such as *Tfrc* and *Slc16a1* were seen, with expression levels peaking toward the capillary–venous end of the spectrum, corresponding to protein expression detected in capillaries and venules but not arteries. Other genes such as *Vwf* and *Vcam1* peaked in arterial and venous clusters, with a loss of expression within the capillary population, whereas *Mfsd2a*, the gene encoding a BBB-specific lipid transporter, was seen to peak in the capillaries, again matching peaks seen in the distribution of protein staining of the marker across different vessel types (Table 1). The study further sought to provide insight on how gene and protein classes were distributed along the arteriovenous axis and, interestingly, found that transcription factor and transmembrane transporter classes showed zonal and nested distributions. Transcription factors were more prominent in arterial clusters, whereas transporter transcripts predominated in capillary and venous populations, indicative of BBB-associated molecular transport functions.

In addition, mural cells were shown in the study to have distinct phenotypic differences across the different vessel types, also displaying arteriovenous zonation. Similar to the endothelial cells, mural cells were grouped into clusters based on transcriptional similarity with the major groupings being pericytes and smooth muscle cells (SMC). SMC populations were further subcategorised as arterial SMC (aSMC), arteriole SMC (aaSMC), and venous SMC (vSMC). One stark contrast to endothelial cell arteriovenous zonation was that the order of transcriptional relatedness of mural cell types (Pericyte → vSMC → aaSMC → aSMC) did not match the anatomical organization along the arteriovenous axis. In fact, two predominant gene expression patterns were found in the mural cell analysis that identified two distinct subclasses of mural cells—one group consisting of pericytes occurring in continuum with vSMCs through gradual loss of pericyte markers and gain of SMC markers, and the other consisting of aaSMCs in continuum with aSMCs through the progressive gain of aSMC markers. High resolution imaging of the SMC marker smooth muscle α-2 actin (ACTA2), and the pericyte marker platelet-derived growth factor receptor beta (PDGFRβ) expression further indicated the abrupt transition between the two major subclasses of mural cells at the arteriole–capillary boundary where arterioles predominant in aaSMC transition to pericytes surrounding the capillaries.

Other scRNA-seq analyses of the brain endothelium have subsequently corroborated the findings of this landmark study [28]. Furthermore, these later studies have transcriptomically characterised additional functionally specialised endothelial subpopulations such as the choroid plexus and meningeal endothelium. This data highlighted the fenestrated characteristics of the cells through identifying high expression of permeability marker genes such as *Plvap* and low expression of tight junction-encoding genes such as *Cldn5* (Table 1) [29,30,31].

### 2.3. CNS Drainage

There also exists the concept of the “third circulation” within the brain. This refers to CSF flowing through the ventricles, cisterns and the subarachnoid space in the meninges, which creates a separate network of fluid flow between chambers and pockets within the cerebral cortex. The ventricles of the brain are a network of cavities that are the main site of CSF transport throughout the CNS. CSF itself is produced by vascularised secretory structures known as the choroid plexus, which are found in each ventricular cavity [32]. The choroid plexus capillary vessels receive their blood supply from the anterior choroidal arteries which branch from the internal carotid artery (Figure 1B). These capillaries are fenestrated (porous), lacking the abundant tight junctions typical within vessels of the BBB, and therefore allowing molecules to more readily diffuse across the vessel walls [33]. CSF arises from the fluid in the capillaries by first passively diffusing across the highly permeable vessels. The fluid then traverses a barrier of ependymal cells which selectively regulate fluid flow into the CSF space under the tight control of transporters such as aquaporins (Figure 1D) [34]. The ependymal cells that line the ventricular cavities contain tight junctions, creating a blood–CSF barrier as opposed to the typical BBB created by the endothelium [32].

Virchow–Robin spaces (VRS) are key sites of fluid exchange between the brain parenchyma and the CSF space. These consist of a network of perivascular spaces or channels formed via separation between the layer of astrocytic foot processes (glial limitans) and endothelial basement membranes [34,35]. Here, CSF diffuses into the parenchyma, and conversely interstitial fluid from the parenchyma drains into the CSF space (Figure 1B) [22]. This fluid flux acts as a waste clearance system for the CNS, eliminating soluble proteins and metabolites from the parenchyma, and is also referred to as the glymphatic system, based on its similarities to lymphatic drainage systems that is present within the meningeal layers of the brain (see below) but absent within the brain parenchyma [36].

The dura is also a major site of venous drainage in the brain, where the superior sagittal sinus, running along the midline of the cortical surface, drains cerebral veins by providing a low-pressure system for blood flow back into systemic circulation (Figure 1A,B) [37,38]. Deep cerebral veins drain from within the cortex into the dural sinuses and back into systemic circulation via the internal jugular veins in the neck (Figure 1A,B) [39,40,41]. CSF drainage also occurs through arachnoid granulations, villi-like projections protruding from the subarachnoid space, which project into the dural sinuses, providing an interface for CSF reabsorption into the venous circulation [34]. In addition, the dura has recently been found to contain lymphatic vessels in close association with the dural sinuses, which were shown to be a potential site for CNS drainage and antigen presentation [30,42,43]. The dural lymphatics were shown to sample macromolecules and immune cells from the CSF and drain into the deep and superficial cervical lymph nodes (Figure 1B) [44]. This is in addition to the already characterised drainage route of CSF from the nasal mucosal lymphatics to the superficial cervical lymph nodes [45].

## 3. The Brain Metastatic Cascade and Tumour Microenvironment

For some time within the field of metastasis, a key area of interest has related to a concept that was first theorized in 1889 termed the “seed and soil” hypothesis, which highlights the intrinsic properties certain sites (“soil”) possess that make them a preferential site for establishment of secondary tumours (“seed”) [46]. This concept begins to explain why certain primary solid tumours have a high affinity towards spreading to a select few organs (organotropism), e.g., prostate cancer spread to the bones [47]. It is thus important to understand in depth the various stages of the metastatic cascade, including the pre-conditioning of future metastatic sites (termed the pre-metastatic niche) via circulating tumour-derived elements, dissemination of cancer cells into blood and lymphatic vessels, extravasation into distant tissue sites, metabolic reprogramming, and the formation of a metastatic niche that supports sustained growth and survival of metastatic lesions.

### 3.1. Clinical Presentation

The ability to detect brain metastases in patients is improving with advances in technology, nevertheless a stark number of brain metastases remain undetected until autopsy analysis in otherwise asymptomatic patients, occurring at rates of up to 75% [48]. This indicates that as systemic therapies, imaging and biopsy technologies improve it is likely that more brain metastases will be observed in late-stage disease of patients with solid primary tumours that show a particularly high affinity to spreading to the brain, such as lung, breast, and melanoma. Magnetic resonance imaging (MRI) is the preferential imaging choice for diagnosis of brain metastasis and remains the gold standard imaging technique for neuro-oncologic practice [49]. Most metastases are thought to arrive at the brain through hematogenous spread through arterial circulation and are most commonly found at the grey and white matter junctions due to the narrowing of blood vessels that leads to cancer cell entrapment and arrest [50,51]. Metastases are typically spherical in shape, with greater incidence in the cerebral hemispheres compared to the cerebellum and brainstem, which approximately follows their relative weights and volumes of blood flow [52,53]. However, small intracranial metastases, representative of early-stage metastatic lesions, have been shown to display different physical characteristics. In a recent study, small intracranial melanoma metastases were observed predominately at the corticomeningeal surface, often presenting as nodular, elongated, or curvilinear metastases presenting with broad contact to the pia mater [54]. In rarer circumstances, patients present with leptomeningeal disease, which involves significant detections of tumour cells within the CSF of the subarachnoid space.

Extensive reviews have already been undertaken on the current treatment modalities and their efficacy for brain metastases from different primary tumour sources [52,55,56,57,58]. The BBB presents perhaps the greatest challenge in developing drugs that are effective for the treatment of brain tumours. Drugs delivered through the circulation are subject to the same constraints provided by the BBB that tightly regulate molecular transport across the endothelial cell layer, through mechanisms such as reduced paracellular transport and endocytosis [6]. A major barrier includes the high affinity that circulating pharmacological compounds have for efflux transporters such as multi-drug resistance ATP-binding cassette (ABC) transporters [59]. These ABC transporters are located on both the luminal and abluminal side of the vessel walls, and primarily function to clear toxins from the parenchyma [60]. The high binding affinity to these transporters can lead to short lived drug activity for compounds that make it into the parenchyma, as active efflux pumps will continue to function to clear them out [16,61]. This is one of the primary reasons that standard-of-care cancer treatments such as chemotherapy often fail to have marked effects on brain metastases, and perhaps why targeted therapies have shown more success [52,62,63,64]. This has been the subject of intense investigation for decades, and many drugs and combinatorial treatments targeted towards the BBB and brain tumour vasculature that have showed promise at the pre-clinical stage have failed to eventuate into clinically effective treatments [65].

Immune therapies, such as checkpoint inhibitors, are possibly the most promising treatment modality for advanced metastases. It has recently been shown that anti-CTLA-4 in combination with anti-PD-1 therapy has resulted in response rates of 45–60% in patients with melanoma brain metastases [66,67]. CTLA-4 is a receptor expressed on the surface of T-cells which competitively binds to CD80/CD86. By this mechanism it acts as an immune checkpoint inhibitor by preventing binding of CD28 to CD80/CD86, thus preventing the co-stimulatory signalling cascade that promotes proliferation and cytokine production and dampening T cell activation [68,69,70]. Blockade of CTLA-4 binding using monoclonal antibodies therefore prevents this dampening, resulting in increased T-cell response and proliferation. PD-1, also a T-cell surface immune checkpoint inhibitor, is present on activated T-cells. Its ligand PD-L1, present on tumour cells and tumour stroma, binds PD-1 on activated tumour-targeting T-cells, which inhibits T-cell proliferation and cytokine production, promoting tumour self-tolerance [68,69,70]. Thus, by inhibiting either PD-1 or PD-L1, a tumour’s ability to promote self-tolerance is hampered. In the case of brain metastases, the promising efficacy of these checkpoint inhibitors suggest that these drugs are potentially increasing immune cell penetration into the CNS as well T-cell activation, reducing tumour self-tolerance. However, the exact biological mechanisms are yet to be fully established [69,71]. Furthermore, anti-PD-1 has been shown in pre-clinical models to be potentiated by VEGF-C through increased VEGFR signalling in meningeal lymphatic endothelial cells, showing how our increased level of understanding of cerebral vascular anatomy and molecular pathways may lead to better targeted therapies toward the brain [72].

The meninges, parenchymal penetrating blood vessels, ventricles, and CSF are all sites of metastatic lesion formation or circulating tumour cell accumulation, each presenting their own challenges regarding treatment [73,74,75,76]. Fluid circulation within the brain is intricately organised and compartmentalised, providing several routes of cell and molecular transport. This in turn provides several routes of potential trafficking for tumour cells, which may seek refuge in favourable niches that are a product of the natural circulatory dynamics and intrinsic stromal cell properties of different vascular microenvironments within the brain. In this context, understanding the key molecular drivers of site-specific cancer spread to the brain from distant solid tumours may elucidate not only targeted treatments toward brain metastases, but also preventative treatments to negate initial metastatic lesion formation.

### 3.2. The Brain Pre-Metastatic Niche and Organotropism

**Figure 2:**
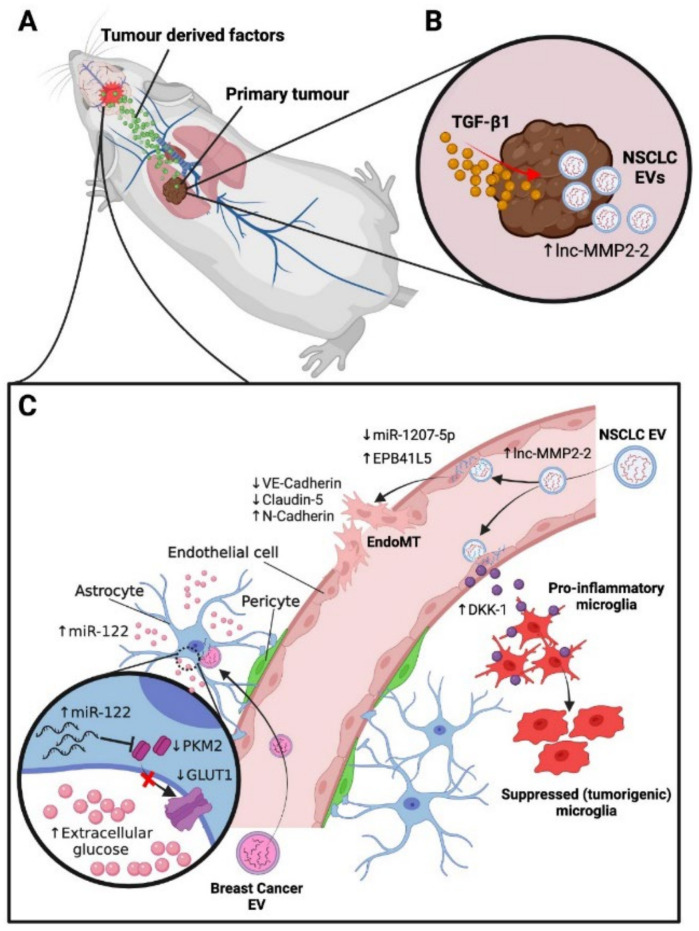
Characterised mechanisms of pre-metastatic niche formation in the brain. (**A**)—Primary tumours secrete soluble factors and extracellular vesicles (EVs) into circulation, which subsequently act on the vascular microenvironments of receptive distant organs to generate pre-metastatic niches. (**B**)—In mouse non-small cell lung cancer (NSCLC) tumours, extensive conditioning by TGF-β1 leads to EV production with increased levels of lnc-MMP2-2 [90]. (**C**)—Top: NSCLC EVs release lnc-MMP2-2 into endothelial cells of the blood–brain barrier, sequestering miR-1207-5p, a suppressor of EPB41L5. Elevated EPB41L5 causes decreased VE-cadherin and claudin-5 expression and increased N-cadherin expression, leading to EndoMT and increased vascular permeability [91,92,93,94]. NSCLC EVs also induce secretion of Dkk-1 by brain endothelial cells, contributing to suppression of pro-inflammatory microglia and increases in tumorigenic microglia, thus promoting an immunosuppressive microenvironment [96]. Bottom: Mouse breast cancer EVs containing high levels of miR-122 suppress glucose uptake by astrocytes by downregulating pyruvate kinase (PKM2) and glucose transport channel GLUT-1. This increases extracellular glucose in the pre-metastatic niche, thereby enhancing glucose availability for tumour cells [97].

In order for circulating tumour cells to successfully seed into distant tissues, they require a favourable environment (“soil”) in which to do so, containing the necessary nutrients, extracellular matrix proteins, and supporting cells to sustain the ongoing growth and proliferation of a metastatic lesion after initial dissemination [77]. It continues to be demonstrated that tumours will in fact induce the formation of these favourable microenvironments, preceding the arrival of circulating tumour cells, via the secretion of soluble tumour-derived factors and extracellular vesicles (EVs) (Figure 2A) [10,78,79,80]. Microenvironmental changes in normal tissue leading to the formation of a pre-metastatic niche include vascular disruption, accumulation of extracellular matrix proteins, and the recruitment and activation of supporting cells such as fibroblasts and various bone marrow-derived cells [81]. Evidently, the pre-metastatic microenvironment is intrinsically tethered to the vasculature, both as a transport network for soluble tumour-derived factors, EVs and cells, as well as being the primary site of pre-metastatic niche formation and tumour-induced changes such as vascular leakiness and remodelling [82,83]. Site-specific tumour cell spread to the brain from primary tumours such as lung cancers, breast cancers and melanoma has been well documented in both humans and experimental models for some time [5,84,85,86]. However, historically, little investigation has been carried out to uncover the intricacies of the brain pre-metastatic niche and how this may drive the organotropic spread observed, and it is only recently that the topic has garnered more attention.

Transforming growth factor-β (TGF-β) is a well-known inducer of epithelial to mesenchymal transition (EMT) and has been implicated in both pre-metastatic niche formation and in promoting the metastatic potential of tumour cells [87,88,89]. In animal models of brain metastasis of non-small cell lung cancer (NSCLC), it was shown that NSCLC-derived exosomes (nanometre-sized EVs that transport proteins, genetic material and other cargo between cells) conditioned by TGF-β1 had increased expression of the long non-coding RNA lnc-MMP2-2 (Figure 2B). This would in turn promote endothelial to mesenchymal transition (EndoMT) in the brain endothelium, a related but different process to EMT, altering the integrity of the BBB prior to tumour cell arrival by downregulating the expression of tight junction proteins and increasing the permeability of the endothelial cell monolayer [90,91] (Figure 2C). It was further demonstrated that the inhibition of lnc-MMP2-2 reversed these effects, and conversely overexpression increased them, doing so by acting as competing endogenous RNA for miR-1207-5p, thus preventing the downregulation of *EPB41L5* mRNA, the encoded protein of which has known oncogenic effects [92,93,94]. It was then demonstrated that EPB41L5 induced EndoMT in vitro by downregulating adhesion and tight junction proteins such as VE-cadherin and Claudin-5, and upregulating mesenchymal markers such as N-Cadherin, thus leading to tight junction disruption and increased BBB permeability [91]. It was finally shown in vivo that lnc-MMP2-2 knockdown significantly inhibited the occurrence of brain metastases, and TGF-β1 pre-treatment of the NSCLC cell line increased the rate of brain metastases. This indicates that a favourable microenvironment was created within the brain for metastatic colonisation, via EV-induced EndoMT.

Conversely, the TGFβ receptor ALK7 has been identified as a suppressor of tumorigenesis and metastasis in a mouse model of breast cancer [95]. By upregulating ALK7 expression in breast cancer cell lines, the size and numbers of metastatic lesions in the lung and brain were significantly reduced. ALK7 activation induces apoptosis in neoplastic cells, and its ligand activin B was found to be ubiquitously expressed in stromal cells of the brain and lung. This essentially forms a paracrine barrier to metastatic colonisation, whereby circulating ALK7+ cells, upon lodging in vascular branches, would be exposed to apoptotic signals by the surrounding vascular stromal cells. Interestingly, it was shown that the stromal cells expressing high levels of the activin B transcript *Inhβb* in the brain were astrocytes as well as a subset of endothelial cells. These endothelial cells were suggested to be more active in protein synthesis due to the abundance of ribosomal transcripts and were postulated to be distributed across all cellular subsets along the arteriovenous axis due to the presence of evenly distributed markers [28]. What regulates the activin B expression in these cells, and whether the endothelial cell subset is distributed evenly across spatial regions of the brain is yet to be investigated, but these findings provide insight into the endothelial subsets that may play an active role in the initial stages of brain metastasis.

Exosomes have also been implicated in a synergistic signalling cascade between brain endothelial cells and microglia of the brain pre-metastatic niche. Animal models of brain metastases showed that upon exosome internalisation of lung cancer cell-derived exosomes, endothelial cells send suppressive signals toward microglia, via the release of endogenous Dkk-1, contributing to the creation of an immunosuppressive environment (Figure 2C) [96]. Conversely, following antibody-mediated blockade of Dkk-1 in metastatic lung cancer cells, microglia returned to their normal, more pro-inflammatory (non-suppressed) states. Metabolic reprogramming has also been demonstrated as another induced change in the cells of the pre-metastatic microenvironment. Breast cancer-secreted EVs were shown to contain high levels of miR-122, which was shown to suppress the glucose uptake of brain stromal cells of the pre-metastatic niche by downregulating pyruvate kinase and the glucose transporter GLUT1, which in turn increased extracellular glucose availability for tumour cells (Figure 2C) [97]. Accordingly, miR-122 inhibition was shown to restore normal glucose metabolism in the brain stromal cell niches and reduce the incidence of metastasis in animal models. A recent proteomic analysis of exosomes derived from a brain-metastasising breast cancer cell line (MDA-MB-231) has shed light on the role of cell migration-inducing and hyaluronan-binding protein (CEMIP) in brain metastasis [11]. CEMIP was one of few highly differentially expressed proteins through quantitative mass spectrometry on exosomes derived from brain-tropic cell lines compared to the parental, lung and bone metastasising variants. Further analysis revealed that CEMIP induces vascular remodelling and inflammation within the brain vascular niche, through upregulation of pro-inflammatory cytokines. In turn, it was shown that CEMIP depletion in the brain impairs brain metastasis formation and the tumour cells’ ability to successfully associate with the brain vasculature. In the human condition, CEMIP was elevated in tissue samples of patients with brain metastases, and CEMIP expression correlated with brain metastases as opposed to other organ metastases, as well as predicting patient survival.

### 3.3. Metastatic Colonisation of the Brain

Hematogenous spread of disseminated tumour cells from a distant primary tumour toward the brain and subsequent micro-metastasis formation is an extremely inefficient process. It has been shown in mouse experimental models of brain metastasis that upon vascular arrest of tumour cells in the brain, approximately 1–5% of cells will actually go on to form a macrometastasis [98,99]. In large part this is due to the difficulty the blood–brain barrier presents for cells to extravasate into the brain for eventual colonisation and secondary tumour formation. Gene expression analysis has shown that α2,6-sialyltransferase ST6GALNAC5 specifically mediates breast cancer metastasis to the brain by enhancing adhesion to the brain endothelial cells and subsequent passage through the blood–brain barrier [100]. Additional mediators found include prostaglandin-synthesizing enzyme cyclooxygenase-2 (COX2) and epidermal growth factor receptor (EGFR) ligands heparin-binding EGF (HBEGF) and epiregulin (EREG). BBB permeability has been shown to increase as a direct consequence of prostaglandin production during inflammation, and HBEGF has been shown to promote tumour cell motility and invasiveness [101,102]. COX2, HBEGF and EREG knockdowns were shown to inhibit transmigration through an in vitro BBB model composed of primary human endothelial cells and astrocytes [100]. This illustrates the reliance that tumour cells have on acquiring mutations that promote blood–brain barrier breakdown, vessel permeability, and invasiveness in order to successfully colonise the brain.

**Figure 3:**
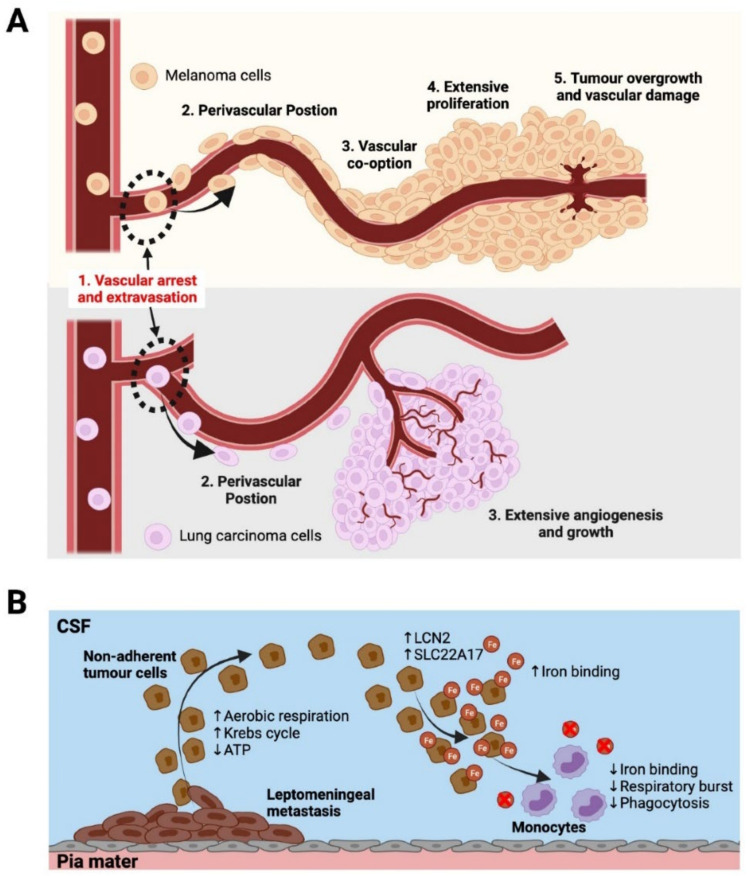
Different modes of metastatic colonisation in brain parenchyma and leptomeninges. (**A**)—Metastatic colonisation of the brain adapted from Kienast et al. [98]: (1) Vascular arrest of cancer cells at narrowing capillaries, and extravasation into the perivascular space. (2) Cells obtain a perivascular position on the abluminal surface of the blood vessels in order to survive and proliferate. (3) Lung carcinoma cells induce marked angiogenesis, attracting an extensive vascular supply and proliferate quickly as a result. In contrast, melanoma cells proliferate along existing blood vessels via vascular co-option in a much slower fashion. (4) Melanoma cells form a growing tumour mass in close association with the existing blood vessels. (5) Tumour overgrowth may lead to vascular damage and bleeding. (**B**)—Leptomeningeal disease. Circulating tumour cells in the CSF of the subarachnoid space are thought to arise from non-adherent tumour cells and exhibit increased aerobic respiration, Krebs cycle and decreased ATP consumption compared to adherent leptomeningeal metastases. These tumour cells also show increased binding of iron through the LCN2/SLC22A17 signalling cascade. This allows them to outcompete monocytes for iron, resulting in decreased respiratory burst and phagocytosis [109,110].

After tumour cell arrest and successful extravasation, tumour cells attempting to colonise the brain require close physical contact to the abluminal surface of blood vessel walls in a pericyte-like position in the perivascular space (Figure 3A) [98]. Melanoma cells, upon attaining their perivascular position, were found to form micro-metastases in close association with existing vasculature in mouse models (vessel co-option), proliferating along microvessels (Figure 3A). These cells would only go on to induce extensive vascular changes and angiogenesis when a large macrometastasis had formed. Conversely, lung carcinoma cells arrested in brain capillaries showed marked early angiogenesis and vascular remodelling, resulting in rapid proliferation to generate a macrometastasis (Figure 3A) [98]. These findings have similarly been reported in humans, with one study demonstrating that out of eight autopsy cases of melanoma brain metastases, all cases showed extensive evidence of co-optive growth along the abluminal surface of blood vessels, displaying features of pericyte mimicry [103]. The study also found extensive vessel dilation and bleeding within the brain lesions, which was consistent with observations in animal models [104]. Investigators postulated that small vessels were being destroyed due to the overgrowth of tumour cells along the vessel surface, which compromised the vessel integrity and lead to marked vascular damage (Figure 3A), a phenomenon that is not often observed in extracerebral sites of metastasis.

It is becoming increasingly evident that to fully understand how metastases colonise the brain, one must take into account the dynamic mechanical and molecular processes governing early tumour establishment, as the location of larger established secondary tumours in the brain may not be representative of the infiltration and early seeding sites. An example has been illustrated in a recent study where it was found that in the majority (up to 90%) of small intracranial melanoma metastases (2–9 mm) in a cohort of patients developed in close relationship to the leptomeninges, commonly at the cortical-pial surface, as well as in the ventricular system and perivascular spaces [54]. It was postulated that the high incidence of small secondary lesions contacting the pial surface indicated a possible preferential site of entry for melanoma cells into the CNS, with extensive parenchymal infiltration occurring secondarily. This detection of early seeding events of melanoma metastases in the leptomeninges prior to parenchymal infiltration was novel in humans and differs from the conventional understanding of how parenchymal brain metastases arise. These events have been well characterised in prior mouse models of brain metastasis, suggesting that investigating site-specific spread of tumours toward the leptomeninges may change our approach to understanding initial seeding events in the brain [105]. B16 melanoma cells were found to have a particular affinity toward the leptomeninges and ventricles upon injection into circulation, with no incidence of parenchymal metastases after 3–4 weeks. Furthermore, mice injected with human melanoma cells isolated from subcutaneous and lymph node metastases primarily developed leptomeningeal metastases; conversely, cells isolated from the parenchyma predominantly produced parenchymal metastases [106,107]. However, these studies have at times been dismissed as clinically irrelevant, due to the apparent low incidence of leptomeningeal metastases observed in patients [108]. The mounting recent and historical evidence demonstrating that the leptomeninges may be a site of interest for the early seeding of metastatic lesions in the brain does however warrant more in-depth characterisation.

Leptomeningeal disease, although less common, is also another significant form of cerebral metastasis that confers poor prognosis, diagnosed by the identification of tumour cells circulating within the CSF. Leptomeningeal disease has been found often in close association with leptomeningeal metastases and is thought to be characterised by a change from an adherent phenotype (typical of leptomeningeal metastases) to a floating, more aggressive phenotype that circulates in the CSF [109]. Through transcriptomic analysis it was shown that floating non-adherent cancer cells had enriched expression of genes involved in aerobic respiration and Krebs cycle and generated less ATP than their adherent counterparts (Figure 3B). The microenvironment within the CSF is quite harsh: tumour cells that find their way into the CSF are subjected to a hypoxic environment with little access to nutrients, which may begin to explain why non-adherent circulating leptomeningeal tumour cells showed key metabolic differences to their adherent counterparts. In addition, it was recently demonstrated that tumour cells in the CSF attain phenotypic changes to allow them to out-compete other cells for iron, by upregulating expression of the iron-binding protein lipocalin-2 (LCN2) and its receptor SLC22A17, which was subsequently shown to promote cancer cell growth in the leptomeningeal space (Figure 3B) [110]. As a result, macrophages (the predominant iron-utilising cell in CSF) became iron-deficient, resulting in impaired oxidative (respiratory) burst and phagocytosis, key components of the innate immune response, thus reducing their immunogenicity, serving as an immune escape mechanism for the cancer cells in the CSF. Furthermore, in animal models of CSF metastasis from lung and breast cancer cell lines, complement 3 (C3) was found to be upregulated and necessary for tumour cell growth in the leptomeninges [111]. C3 was shown to activate the C3a receptor in the ependymal cells that line the choroid plexus, disrupting the blood–CSF barrier and allowing mitogens from the plasma to enter the CSF, promoting cell proliferation and growth.

### 3.4. Tumour Drainage

Recent advances in characterising the meningeal lymphatic system have illustrated an avenue for soluble factors and immune cells to drain from CSF within the subarachnoid space into the cervical lymph nodes [42,43]. It was demonstrated that endothelial and mural cells within the dural sinuses enabled a previously uncharacterised CNS surveillance system that involved antigen accumulation and presentation at key sites around the dural sinuses in close relation to the lymphatic vessels [30]. Furthermore, in mouse models it has been shown that ageing is associated with impaired meningeal lymphatic function, leading to accelerated accumulation of the toxic protein amyloid beta, a hallmark of Alzheimer’s disease [112]. It was further shown that vascular endothelial growth factor C (VEGF-C) treatment potentiated immunotherapies against amyloid beta, leading to better outcomes in mouse models of Alzheimer’s disease, elucidating the important roles that the meningeal lymphatic system may play in neurological disease [29]. This was in direct contrast to the extensively characterised historical theory of the brain being an immune-privileged site, due to the necessity of preventing large inflammatory events in the brain that could cause extensive neural tissue damage [113]. These discoveries prompted recent investigations into whether meningeal lymphatics play a role in tumour cell drainage from cerebral metastases. RNA-seq analysis of meningeal lymphatic endothelial cells in mouse models of intracranial glioma and melanoma metastases revealed changes in gene expression of key gene sets involved in lymphatic remodelling, drainage, and immune responses [114]. Furthermore, disruption of the lymphatic vessels impaired intratumor fluid drainage and tumour cell dissemination into the cervical lymph nodes. The study also demonstrated that VEGF-C-overexpressing tumour cells responded far better than controls to checkpoint inhibitor therapy for intracranial tumours, and that this effect was abolished in mice with defective meningeal lymphatics. In a separate study involving mouse models of glioblastoma, it was found that VEGF-C potentiates immune responses against tumours in the brain, and that it was dependent on an intact meningeal lymphatic system [72]. It was shown that VEGF-C promoted T-cell priming in the deep cervical lymph nodes draining the tumour and increased the migration of cytotoxic T-cells toward the tumour. These effects were then shown to be reversed when the meningeal lymphatic vessels were ligated. They further illustrated that pre-treatment of mice with VEGF-C in combination with checkpoint inhibitor therapy resulted in the complete rejection of glioblastoma cells. Whilst our understanding of the meningeal lymphatics is far from complete, these new findings pave the way for more studies looking at how the lymphatics play an active role in tumour cell clearance from the brain and potentiate immune responses against brain cancers.

## 4. Future Research and Conclusions

Understanding tumour–vasculature interactions at the early stages of tumour formation as well as before tumour cells have established is key for progressing our understanding of organotropic tumour cell spread. The pre-metastatic niche is becoming a critical point of interest in cancer metastasis research and understanding the mechanisms regulating its formation could play a vital role in the future for creating preventative treatments. Extracellular vesicles have been shown to play a major role in shaping pre-metastatic environments, and influencing endothelial cell behaviour [11,28,87,88,89,90,91,92,93,94,95,96,97]. In addition, early metastatic colonisation of the brain has been shown to be intricately coupled to tumour cell positioning along blood vessel surfaces during vascular arrest and extravasation, further highlighting the importance of vascular biology at various stages of metastatic progression [98]. Specific diagnostic markers that indicate the shaping of favourable pre-metastatic environments that may spatially predict future metastatic sites in the brain are yet to be elucidated. Large-scale discovery experiments using technologies such as single-cell RNA sequencing (scRNA-seq), may provide researchers with new avenues to seek and discover these markers.

ScRNA-seq has recently come to the forefront of cancer biology research, providing a platform to run large discovery studies on gene expression changes within different disease models at single-cell resolution [115]. This high-throughput sequencing technique, where single cells are isolated and transcripts are captured for each individual cell, boosts the resolution at which we can now analyse transcripts in a heterogenous sample of cells [116]. This becomes very important when aiming to understand the changes in gene expression of particular subsets of cells in a disease model. Combining the use of mouse tumour models of metastatic progression with scRNA-seq analysis can provide a powerful unbiased discovery technique for identifying candidate genes that are implicated in tumour metastasis and growth, potentially shedding light on novel biological mechanisms underpinning complex processes such as dissemination, spread and extravasation. ScRNA-seq has already been implemented in studies attempting to characterize the transcriptomes of brain and meningeal endothelial and stromal cell populations [28,30,31]. However, scRNA-seq studies on endothelial cells in brain metastasis models are yet to be conducted. Some studies have begun using scRNA-seq to characterise molecular changes of endothelial cells in mouse models of glioblastoma as well as in human glioblastoma tissue samples [117,118]. One recent study in a mouse model of glioblastoma identified a rare population of tumour-derived endothelial cells that had differentiated from tumour cells and were molecularly distinct from tumour-associated endothelial cells [118]. Another study characterised the transcriptional profiles of metastasis-associated myeloid cells in the brain in a lung cancer model, illustrating the progressive changes they enact upon the tumour microenvironment [119].

Spatial transcriptomics is another related technique that is beginning to approach the forefront of research into the tumour microenvironment. This technology allows investigators to map transcriptomic expression spatially across a tissue sample, which is extremely useful for visualising tumour–microenvironment interactions. Using a zebrafish model of melanoma, one study uncovered a distinct interface cell state between the tumour boundary and microenvironment cells, where cilia genes were upregulated. Whilst recent studies have implicated cilia in different aspects of melanoma biology, including the deconstruction of cilia driving metastasis, this study was the first to show spatial specificity to the upregulation of cilia genes. More importantly, only cells at the tumour boundary had this high expression, illustrating the power of spatial transcriptomics in coupling spatial and molecular alterations in disease models [120].

It is also important to understand the route of cancer cell spread towards the brain. Particularly the alterations to blood and lymphatic vessels associated with the primary tumours and tumour-draining lymph nodes that allow a cancer to metastasise more readily and promote aggressive phenotypes. It has already been shown that melanoma tumour cells isolated from human lymph nodes will metastasise more readily to the leptomeninges in mouse models, as opposed to tumour cells isolated from the brain parenchyma which produce parenchymal metastases [106,107]. Studies have definitively shown that cancer cells’ ability to metastasise is significantly promoted by lymphangiogenic growth factors VEGF-C and VEGF-D, that both remodel and induce growth of new lymphatic vessels [121,122,123,124,125]. It has also been shown that tumour cells will undergo extensive reprogramming within lymph nodes, creating a more aggressive phenotype that allows them to escape immune responses and protects them from programmed mechanisms of cell death such as ferroptosis [126]. Understanding these processes may help uncover the molecular mechanisms governing organotropic tumour cell spread towards key tissues such as the brain, and how anti-angiogenic therapies may be better incorporated into treatment protocols [127].

It is important to factor in the complexity and heterogeneity of the vascular environments within the brain when designing experimental approaches to understanding tumour–vascular interactions and the brain tumour microenvironment in general. Taking the aforementioned principles into account may lead to interesting experimental designs that seek to characterise the differences in tumour cell colonisation of the brain in different experimental models of metastasis in order to better understand the site-specific spread of cancer to the brain from solid primary tumours. By using new discovery techniques such as scRNA-seq and spatial transcriptomics, more drivers of cancer cell spread to key organs such as the brain will be uncovered. These discoveries will lead to better understanding of cancer cell biology, thereby informing the generation of new therapeutics, more effective use of existing treatments and, ultimately, better outcomes for cancer patients.

## Figures and Tables

**Table 1:** Molecular and transcriptomic markers of brain and meningeal endothelial cells [28,29,30,31].

Endothelial Cell Specialization	Arterial	Capillary	Venous	Choroid Plexus	Meningeal	Meningeal Lymphatic
BBB characteristics	Yes	Yes	Yes	No	No	No
Molecular/transcriptomic markers	*Pecam1* *Cldn5* *Cd34* *Bmx* *Vcam1* *Vegfc* *Efnb2* *Sema3g*	*Pecam1* *Cldn5* *Cd34* *Tfrc* *Mfsd2a* *Slc16a1*	*Pecam1* *Cldn5* *Cd34* *Vwf* *Vcam1* *Slc38a5* *Nr2f2*	*Pecam1* *Cd34* *Plvap* *Plpp3* *Esm1* *Cd24a*	*Pecam1* *Cd34* *Plvap*	*Pecam1* *Lyve1* *Flt4* *Ccl21b* *Prox1*
